# Deep learning early stopping for non-degenerate ghost imaging

**DOI:** 10.1038/s41598-021-88197-5

**Published:** 2021-04-20

**Authors:** Chané Moodley, Bereneice Sephton, Valeria Rodríguez-Fajardo, Andrew Forbes

**Affiliations:** School of Physics, University of the Witwatersrand, Johannesburg, 2000 South Africa

**Keywords:** Quantum optics, Single photons and quantum effects

## Abstract

Quantum ghost imaging offers many advantages over classical imaging, including the ability to probe an object with one wavelength and record the image with another (non-degenerate ghost imaging), but suffers from slow image reconstruction due to sparsity and probabilistic arrival positions of photons. Here, we propose a two-step deep learning approach to establish an optimal early stopping point based on object recognition, even for sparsely filled images. In step one we enhance the reconstructed image after every measurement by a deep convolutional auto-encoder, followed by step two in which a classifier is used to recognise the image. We test this approach on a non-degenerate ghost imaging setup while varying physical parameters such as the mask type and resolution. We achieved a fivefold decrease in image acquisition time at a recognition confidence of $75\%$. The significant reduction in experimental running time is an important step towards real-time ghost imaging, as well as object recognition with few photons, e.g., in the detection of light sensitive structures.

## Introduction

Ghost imaging is a technique where the correlations between two spatially separated photons are used to reconstruct an image of an object. Neither photon alone has complete information about the object: the object photon is collected with a detector without any spatial information, while the imaging photon has not physically interacted with the object^1, 2^. Individually each photon cannot offer any image information retrieval however the correlations between them allow for image acquisition^3, 4^. While originally demonstrated as a quantum entanglement phenomenon^5^, it was later shown that ghost imaging can also occur as a result of classical correlations^6–8^. Quantum ghost imaging was initially thought to produce higher resolution images, however we now know that both methods produce images of a similar resolution^9, 10^. Advantageously, the use of quantum light allows for imaging at low light levels, demonstrating a higher signal to noise ratio and visibility^9, 11^. Degenerate quantum ghost imaging systems consist of signal and idler photons that are of the same wavelength, it is however possible to implement a non-degenerate ghost imaging system in which the signal and idler photons are of different wavelengths^12, 13^. A non-degenerate system enables the imaging of objects within certain wavelength bandwidths where spatially resolved detectors are impractical or ineffective in terms of resolution. As with the degenerate case, non-degenerate ghost imaging does not offer any resolution improvement over systems using classical correlations^14^, and the spatial resolution of the image depends primarily on the wavelength which illuminates it^15^. Particularly, quantum imaging is useful for biological imaging applications where it is beneficial to reduce the risk of photo-damage^16^.

Furthermore a limitation that ghost imaging faces is the unsatisfactory imaging speeds. Earlier raster scanning implementations were used^5^, which evolved to more timely methods using a single-pixel bucket detector and pre-computed binary intensity fluctuation patterns^17^, single-pixel scanning methods^18, 19^ and Fourier single-pixel scanning methods^20^. With each new method, the imaging speed remained unsatisfactory and so too did the number of measurements required to image the object^21, 22^. Attempts to improve the imaging speed focused on improving the method of data acquisition^23, 24^, the data processing method such as compressive sensing^22, 25, 26^, image reconstruction algorithms such as the fast Walsh-Hadamard transform^27^ and logarithmic and exponential ghost imaging reconstruction algorithms^21^. The use of machine learning algorithms has recently gained a lot of interest due to their promising capabilities. Deep learning has been used to increase image reconstruction quality^28^ and improve image quality by denoising mechanisms^29, 30^ through the use of neural networks. To speed up and enhance image reconstructions deep convolution auto-encoders have been used as a self-supervised learning approach^31, 32^. In cases where object discrimination is of primary importance recognition algorithms have been used to decrease image reconstruction time^33^.

In this work we combined two powerful machine learning algorithms in a two-step deep learning approach to reduce image reconstruction time by establishing an optimal early stopping point for the experiment. We harnessed the power of a combination of self-supervised and supervised machine learning algorithms to reduce the number of measurements required to reconstruct the image while still preserving all relevant object information. Our two-step approach leverages a deep convolutional auto-encoder network to enhance the reconstructed image after each measurement and a recognition algorithm to predict the confidence level of object recognition after each measurement. Here, we start by describing the strategy, followed by the implementation details and finally present our results, where we demonstrate a significant reduction in the time necessary for image reconstruction. We believe that this novel two-step deep learning approach will provide valuable insight into early stopping methods for ghost imaging experiments while preserving image information through a combined process of image enhancement and recognition.

## Strategy

**Figure 1:**
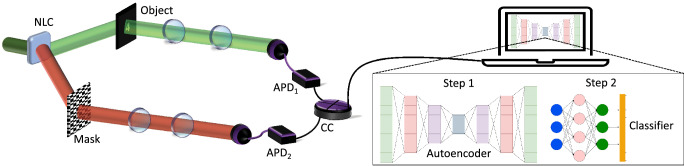
Conceptual sketch of an all-digital ghost imaging setup powered by a two-step deep learning approach. Non-degenerate entangled photons are spatially separated along two arms. One photon interacts with the object and is collected by a bucket detector. The other photon is collected by a spatially resolving detector comprising a patterned mask and a bucket detector. Each detector is connected to a coincidence counting device to perform coincidence measurements. The image is reconstructed by a linear combination of the patterned masks weighted by the coincidences. The reconstructed image, after each measurement, is passed through both the deep learning algorithms for image enhancement and recognition.

Figure 1 illustrates conceptually our non-degenerate all-digital ghost imaging setup powered by a two-step deep learning approach. Here, the required position correlations in the quantum regime arise from the spatial entanglement of the signal and idler photons produced by a non-degenerate spontaneous parametric downconversion (SPDC) process using a non-linear crystal (NLC)^34^, i.e., the signal and idler photons are not of the same wavelength. The entangled photon pairs are spatially separated into two independent paths, one to illuminate the object (object arm) and one which is collected by a spatially resolving detector (reference arm). Our spatially resolving detector is accomplished by displaying a series of binary patterns (masks) on a spatial light modulator (SLM) and collecting the projection using a bucket detector^25^. Imperatively, the patterns (or masks) must constitute a complete basis to acquire a fully reconstructed image. Combining a patterned mask with a bucket detector not only constitutes a spatially resolving detector but is also a similar approach to single-pixel imaging. The measured correlations then provide information on the similarity (or overlap) between the object and each patterned mask. The image is reconstructed as a linear combination of all masks weighted by the measured correlations. As with single-pixel scanning systems, scanning through a series of masks results in a very lengthy image reconstruction process which is not optimal and a great area of interest within the ghost imaging community^28, 31, 35, 36^.

**Figure 2:**
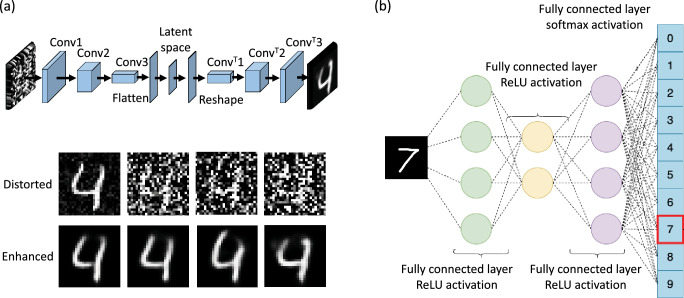
Schematic diagram of the deep learning architectures used. (**a**) The architecture of the deep convolutional auto-encoder showing the convolutional and deconvolution layers used. Images below show steps of dataset distortion achieved by varying a hyper-parameter until the dataset was no longer accurately recognised. The corresponding auto-encoder enhanced counterparts are shown directly below. (**b**) The architecture of the classifier showing each of the fully connected layers with the relative activation functions used in each layer.

We designed and implemented a two-step deep learning approach to reduce the image reconstruction time by establishing an optimal early stopping point for the experiment. The first step is dedicated to enhance the image through denoising the image acquired after each measurement, while the second step recognises the enhanced image. Both steps utilise the power of two different deep neural network architectures as shown in Fig. 2.

In our unsupervised machine learning algorithm, step one, we train an auto-encoder network to enhance the reconstructed image after every measurement by a denoising mechanism. Auto-encoders are built with the practicality of image denoising, reconstruction and data compression and decompression. These neural networks are also used in principle component analysis as a dimensionality reduction technique and can also be used to generate higher resolution images. Auto-encoders are a type of neural network which compress the input into a latent-space representation (also known as a bottleneck). The output is then reconstructed from the latent-space representation, thereby making auto-encoders an unsupervised, or more specifically a self-supervised, machine learning algorithm. An auto-encoder can also be thought of as a feature extraction algorithm in which the output is produced by the features learnt during training. A limitation that exists with auto-encoder networks is their decreased capacity to generalise their output. These particular networks generally enhance images according to what they have already learnt, thereby denoising an output to an image closer to the data they have learnt than the actual object. We implemented a deep convolutional auto-encoder network to encode (compress) and decode (decompress) the ghost imaging object reconstructions after each measurement, the architecture of which can be seen in Fig. 2a. To train this network we used the publicly available MNIST handwritten digit dataset which consists of 70,000 images in a 6 : 1 train-test ratio split. The dataset was distorted by the addition of random noise as shown in Fig. 2a, drawn from a normal distribution, to the point at which a deep learning classifier could no longer accurately recognise any image within the dataset. The distorted images together with their unaltered counterparts were then used to train the network. The encoder extracts the noise to learn a more robust representation of the data, while the decoder will then output a representation sans noise. Our deep convolutional auto-encoder comprises a series of convolutional, activation and pooling layers tailored to map the input features to the output features. This step was employed to enhance the image acquired after each measurement, by a denoising mechanism, before passing the output into our recognition algorithm.

In certain applications the ability to discriminate between different objects outweighs the need for aesthetic similarity between object and image. We, therefore, implemented a deep learning classifier as the second step to establish an early stopping point, the architecture of which can be seen in Fig. 2b. The classifier was trained on the same MNIST dataset in a supervised manner. The labels for this dataset are numerical values however it was important for us to treat the labels as a set of values rather than ordinal numbers. To address this issue with categorical data we encoded the labels into a one-hot encoding vector whose length is equivalent to the number of categories (ten digits therefore ten categories). A one-hot encoding forces the model to select exactly one of the positions in the vector, the output is then the position with the highest predicted probability, i.e. the confidence. Each position within the vector corresponds to a single digit. Tuning the various hyper-parameters to prevent problems such as vanishing/exploding gradient or over-fitting we achieved an acceptable model accuracy. It is important to note that the predicted probability, or the confidence, is not the same as the model accuracy. A $97\%$ model accuracy implies that given 100 images the model will, on average, accurately predict the category of 97 of these with a probability (confidence) that varies largely of anything between 50 and 99%. In our approach, we predicted the category (class) with the highest probability and checked to see if it corresponds to the correct digit, while also considering any close lying probabilities of the other classes for accurate object discrimination. For such a classification problem with 10 categories a predicted probability greater than 50% for a certain category (the sum of the remaining classes is less than half of the total probability) is traditionally acceptable. When assessing the stopping point of the approach, we chose to impose a stricter criteria of a probability of $75\%$, opposed to $50\%$, to stop the experiment.

## Implementation details

**Figure 3:**
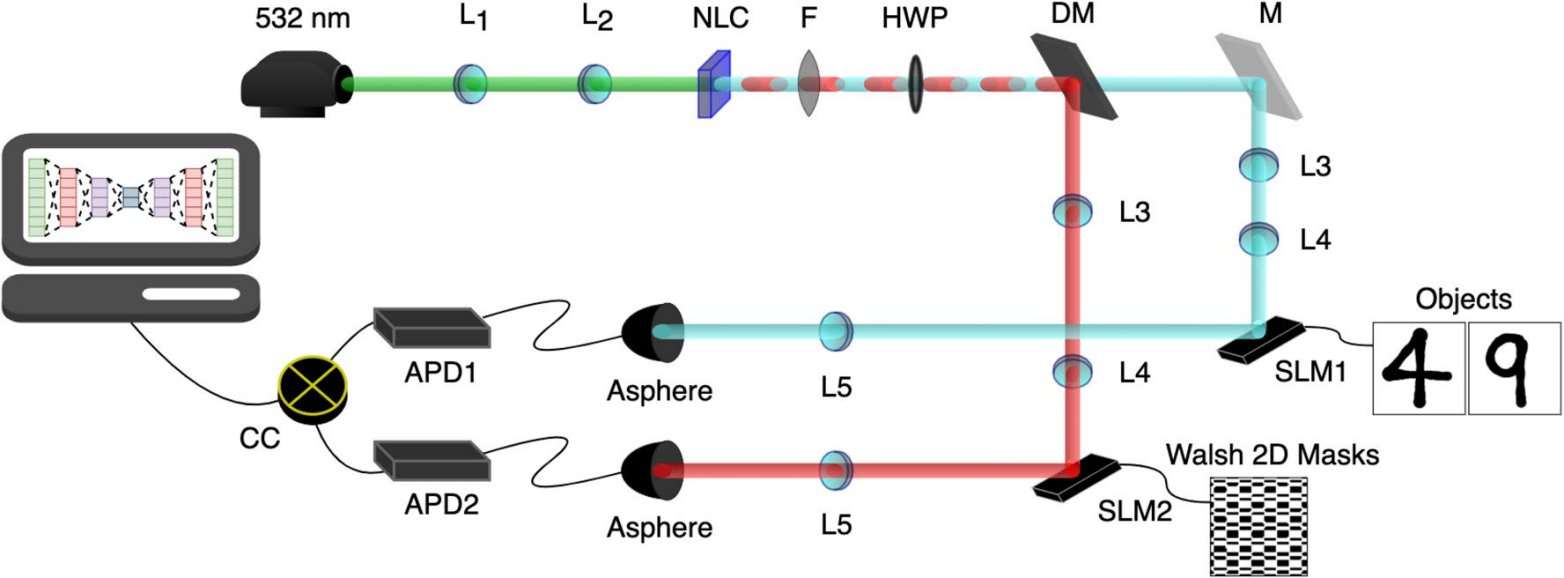
Schematic of the implemented optical setup. Non-degenerate entangled photons are produced at the non-linear crystal (NLC). A dichroic mirror (DM) is used to spatially separate the entangled photons. Each photon impinges on a SLM displaying either the object or patterned mask. The photons are collected by coupling each beam to a multimode fibre connected to an APD, coincidences are then counted to allow for image reconstruction.

Figure 3 shows a schematic representation of the optical setup we implemented in this work. A horizontally polarised laser beam (wavelength of $\lambda = 532\,\hbox {nm}$) was used to pump a type 1 PPKTP non-linear crystal (NLC), whose temperature was set to obtain colinear emission of entangled non-degenerate photons at wavelengths of 1550 nm and 810 nm by a SPDC process. A bandpass filter was used to filter out any non-converted photons. The NLC converted the SPDC photons to a vertical polarisation so a half-waveplate (HWP) was introduced to rotate the light back to a horizontal polarisation for optimal modulation by the spatial light modulators (SLMs). A dichroic mirror was used to spatially separate the non-degenerate entangled photons allowing infrared (1550 nm) transmission while reflecting any near-infrared (810 nm) photons. Infrared photons were directed to the SLM displaying holograms of our chosen objects to be imaged, while the near-infrared photons impinge on the second SLM displaying holograms of our mask patterns. A blazed grating was added to each hologram to separate the first diffracted order from that of the zeroth order which is unmodulated. The modulated light from the first diffracted order was then coupled into multimode optical fibres connected to avalanche photo-diodes (APDs) for single photon-detection. Coincidences were measured by a photon coincidence counter (CC). The raw image was reconstructed by a linear combination of the mask patterns in the chosen basis, weighted by the measured coincidences. Our holograms (objects and masks) were created using MATLAB, for data acquisition we used LabView, all image processing techniques were carried out in JupyterLab using Python.

Network structures of both the auto-encoder and classifier are shown in Fig. 2. In the case of the auto-encoder, for the encoder section we used a three layer 2D convolutional network activated by the ReLU function, which are best suited for handling image data. Each convolutional layer was separated by a maximum pooling layer. Finally the data were flattened and sent to a dense layer activated by the softmax function. Similarly, the decoder section contained a three layer transpose 2D convolutional network, with each layer separated by a batch normalisation layer. Together, both the encoder and decoder were compiled and trained using different optimisers (hyper-parameters). We tested the optimisers Adagrad, AdaDelta and Adam, and settled on using the latter in combination with the loss function as binary cross entropy since this combination provided the best results. In the case of our classifier, it consists of a three layer fully-connected neural network with a softmax output. While the loss function and metric remained fixed, we varied the optimiser to achieve the most promising results. Here our loss function was categorical cross entropy, our metric was accuracy and the optimiser we finally settled on was Adam as this provided the best results, as with the auto-encoder.

While we focused considerable efforts on adequate training and hyper-parameter tuning of the deep neural networks, we also varied physical parameters within the experiment to test the robustness of our deep learning approach. Two such parameters were the patterned mask set type and the mask resolution. For the patterned masks we looked at binary sets of Walsh 2D and random masks as detailed in some of our previous work^33^. Random patterns, since they are not correlated to each other, form an over-complete set with randomly distributed binary pixels, each with a 50% probability of being either black or white. Our Walsh 2D masks are generated by extracting the Walsh functions from the native MATLAB function for Hadamard matrices where *N* is the order or the Hadamard matrix. By performing the outer product between columns of a Hadamard matrix of order *N*, the result is a complete set of $N^2$ Walsh 2D masks of $N\times N$ pixel resolution. Choosing the order of the Hadamard matrix will therefore result in the corresponding mask resolution. The mask resolution (known as speckle spatial size in classical experiments) in turn determines how well the object will be resolved^37^. A higher resolution results in a larger number of basis elements and therefore an increased number of masks is needed to resolve the image. Increasing the resolution has direct consequences on the reconstruction time, the number of Walsh 2D masks required to form a complete set scales as $N^2$ and so there is a trade-off between reconstruction time and image resolution. Ghost imaging object reconstructions were carried out for objects, mask types and resolutions as summarised in Table 1.

**Table 1:** Summary of experimental parameters that were varied during the experiment.

Object	Mask type	Resolution (pixels)
Number 4	Walsh 2D	48 $\times$ 48
Number 9	Walsh 2D	48 $\times$ 48
Number 9	Random	48 $\times$ 48
Number 9	Walsh 2D	96 $\times$ 96

We used digits as objects with the idea of exploiting the considerable development of digit recognition by neural networks. Specifically, we know that neural networks tend to confuse digits four and nine, and so they were chosen to verify the object recognition part of the two-step process. Our neural networks were independently built and trained. Starting with the neural recognition algorithm, we built and trained a neural classifier on the MNIST handwritten digits dataset. We optimised the hyper-parameters, tested on a subset of 10,000 images that were not part of the training set, and obtained a $97\%$ model accuracy. We then moved on to training the auto-encoder for the image enhancement step. Gaussian noise was added to the MNIST dataset with the goal of achieving a distorted dataset that could not be recognised by our recognition algorithm. A hyper-parameter which controlled the level of noise added to the dataset was introduced and increased until the recognition algorithm incorrectly predicted 100% of the dataset. The distorted dataset, in conjunction with its original counterparts, was then used to train the deep convolutional auto-encoder network. The auto-encoder network was optimised by hyper-parameter tuning to enhance the images through a denoising mechanism. Once an acceptable train-test loss was achieved for the auto-encoder network, we passed through our unseen simulated ghost images as a test of the algorithm’s capabilities. Our neural networks were built in JupyterLab using Python with both TensorFlow and Keras as the deep learning frameworks. We trained and optimised our networks pre-measurement, using data from ghost imaging simulations done in MATLAB to further test the capabilities of the networks. For the deep convolutional auto-encoder network the train and test loss showed good agreement indicating that the model was neither under- nor over-fitting. A similar loss was seen for the classifier where the train and test accuracy showed a $1\%$ difference. Most of the computational load was, therefore, shifted to a pre-measurement step.

After each measurement (patterned mask) we applied our two-step approach which consists of passing the reconstructed raw image through our auto-encoder for image enhancement by denoising, followed by passing the enhanced image into our neural classifier for object recognition. The output values of the neural classifier were the predicted digit and its corresponding confidence. For categorical classification such as our recognition step, a probability of $50\%$ or higher is usually considered acceptable (as the sum of the probability of the other nine categories will be $50\%$). We imposed an even stricter evaluation criteria, stating that the recognition algorithm requires a $75\%$ or higher confidence (predicted probability) of accurate image recognition. We chose this confidence stopping point based on the varied probability with which a digit is predicted. A confidence of $75\%$ is therefore in the middle: not as lenient as $50\%$ and not as strict as $99\%$, but still on the higher range of predicted probabilities, imposing a condition for early stopping. Although a confidence of $75\%$ can occur at multiple reconstructions, here we find the optimal stopping point at the first instance of the confidence at $75\%$, making sure that the five preceding reconstructions average to a confidence of above $74.8\%$. At this point, we have achieved image enhancement and recognition and can, therefore, safely say that the experiment can be stopped.

## Results

It was important for us to shift the bulk of the computational load to a pre-measurement step, so that the image enhancement and recognition post-measurement utilise as little time as possible as the goal is to establish the optimal early stopping point. We varied the experimental parameters as aforementioned and saved the raw reconstructed images after each iteration, i.e. after each patterned mask weighted by the coincidences. This was done for both the Walsh 2D and random masks, additionally for the Walsh 2D masks, we tested different resolutions. The goal being to show that our two-step approach can handle different patterned masks and resolutions. We used digits as objects to take advantage of the extensive work done on digit recognition by neural networks. The numbers four and nine were chosen given their non-symmetrical nature^33^. We also know that numbers four and nine are two very similarly written objects, prone to discrimination problems in machines, and therefore we further tested the ability of the two-step approach to accurately discriminate between these digits.

**Figure 4:**
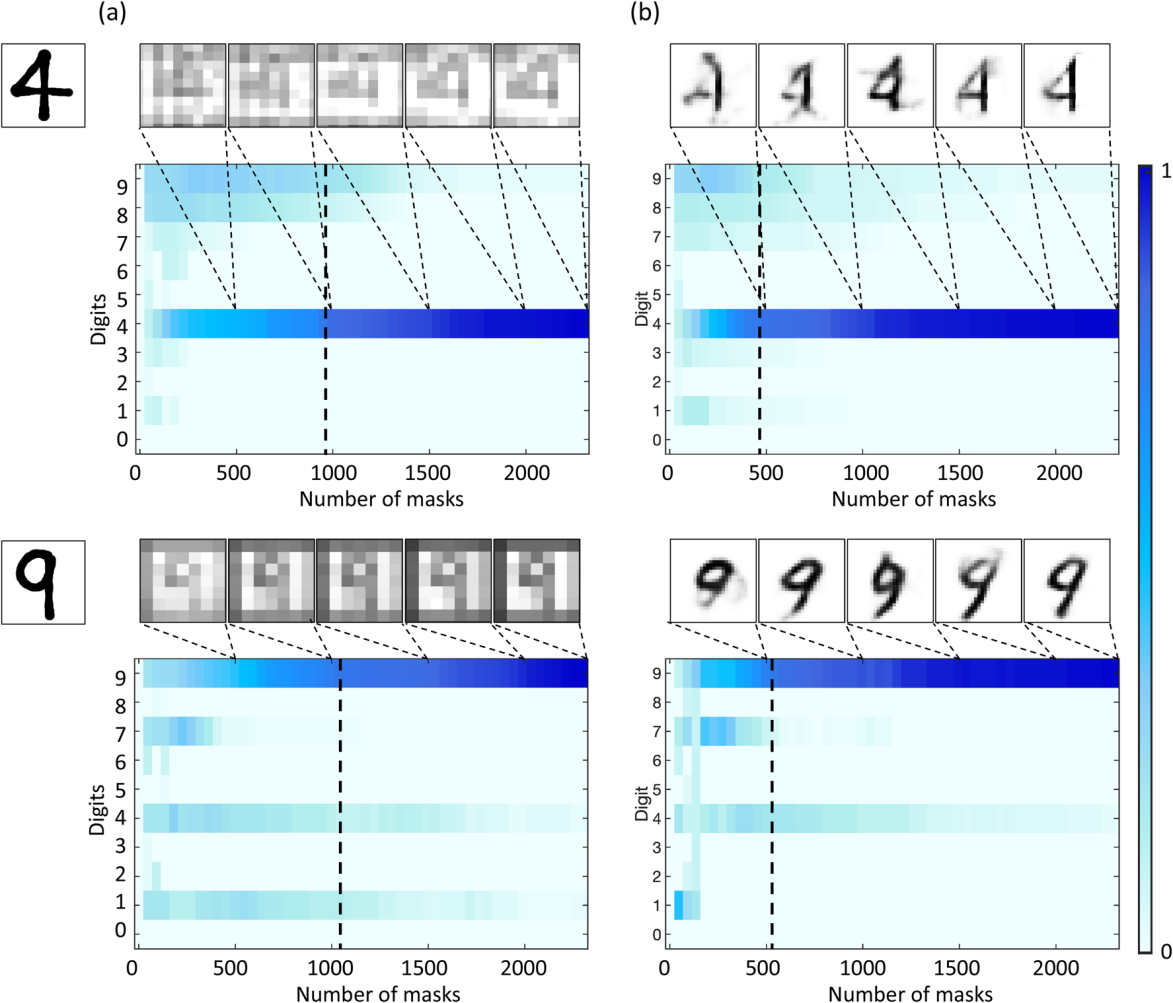
Results of the deep learning recognition algorithm and the two-step deep learning approach. (**a**) The reconstructed raw images for objects four and nine at 20, 40, 60, 80 and $100\%$ image reconstruction time, respectively. Followed by the corresponding confidence predictions for all digits and iterations. (**b**) The aforementioned reconstructed raw images are then passed through the auto-encoder for image enhancement, displayed are the corresponding enhanced images at the same reconstruction times. Followed by the corresponding confidence predictions for all digits and iterations. A significant decrease in the reconstruction time between both cases is shown by the vertical dashed lines.

We first compared the use of a recognition algorithm on its own (one-step) to that of our two-step deep learning approach for digits four and nine as objects. Figure 4 shows the images reconstructed by a complete set of $48 \times 48$ pixel Walsh 2D masks for objects four and nine. All objects in question were designed to be smaller than the imaging area to ensure an even illumination, leading to the effective resolution of the object to be lower than that of the mask. Additionally, for the sake of better visualisation, the images were cropped in all cases. The images displayed are the raw (Fig. 4a) and enhanced (Fig. 4b) images reconstructed at 20, 40, 60, 80 and $100\%$ of the total number of masks, respectively, and the corresponding confidence predictions for all digits and iterations. In the case of the enhanced images, the purpose of our auto-encoder is to enhance the quality of the noisy or raw image. In our case the network enhanced the image through increasing the quality and the signal-to-noise ratio by generalising to what it had previously learnt from the training data, which had better resolution than our experimental images. This type of behaviour is typical to auto-encoders, since they exhibit feature extraction qualities. In Fig. 4 we can see that there are several other digits that come through as strong predictions at low numbers of patterned masks. However, there is a steady and accurate confidence decrease for these digits. Importantly, the confidence of the reconstructed image generally increases in proportion to the number of masks used. The confidence increase is a general trend and confidence fluctuations between each reconstruction exist of less than $0.0005\%$ on average. The general confidence increase validates that the image is recognised with increasing confidence which allows for an early stopping point to be established. We would, therefore, want to reduce the confidence level to as low as acceptably possible without compromising the image information. Vertical dashed lines depict $75\%$ confidence level, which we impose as our criteria for the stopping point. Using a one-step recognition algorithm, Fig. 4a shows that we reach this criteria around 42–45% of the total image reconstruction time, which in itself is a notable reduction. However, when we impose the same criteria on the two-step approach we see a significant shift in the image reconstruction to approximately 20–22% of the total reconstruction time. Importantly, this is not a special case and occurs with all the objects we tested using these experimental parameters, here we display the results for objects numbers four and nine. Not only have we managed to identify the object being imaged with a $75\%$ confidence in object discrimination, but we have also managed to enhance the image to preserve aesthetic similarity between object and image. By preserving visual similarity this means that not only can a computer correctly recognise the image but so too can a human.

**Figure 5:**
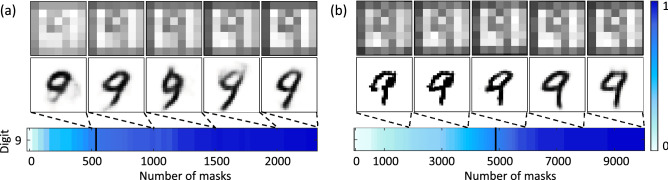
Comparison of the two-step approach for Walsh 2D and Random masks. (**a**) The reconstructed images at 20, 40, 60, 80 and 100% image reconstruction time respectively, for 48 $\times$ 48 pixel Walsh 2D masks. Followed by the corresponding enhanced images from the auto-encoder output. The confidence level, predicted by the recognition algorithm after enhancement, only for digit 9 is shown beneath. (**b**) Here we show the same aspects as (**a**) however 10,000 random masks were used in comparison to the Walsh 2D masks. The solid black lines show the imposed stopping point for each case. It can be seen that the Walsh 2D masks converge $\times$ 10 faster than the random masks.

We further tested the ability of the two-step approach on the random pattern masks. Figure 5 compares the results obtained from the two-step approach when using $48\times 48$ pixel Walsh 2D (Fig. 5a), random (Fig. 5b) masks to reconstruct the image. The images shown are the raw reconstructed images (top row), the auto-encoder enhanced images (middle row) and the corresponding confidence level for this particular object (bottom row). For random masks, as they do not have correlations between them, we used 10,000 masks to achieve an over-complete set. In Fig. 5b we show that random masks achieve the specified criteria at around mask number 4827 after the two-step approach, compared to 5988 masks (not shown here for the sake of compactness) with the use of a recognition algorithm on its own (one-step). Importantly showing that when random masks are used, the two-step approach does indeed reduce image acquisition time. The two-step deep learning approach can, therefore, be applied to different mask types to reduce image acquisition time. In addition, it can be seen that the Walsh 2D masks converge approximately 10$\times$ faster than the random masks, suggesting it would be optimal to impose mask-specific criteria to establish early stopping. For Walsh 2D masks we can confidently stop the experiment around 20% of the reconstruction time, while for the random masks a different criteria would need to be established between 48 and 50% of 10,000 masks.

**Figure 6:**
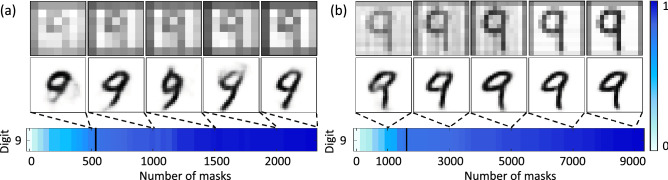
Comparison of the two step approach for different resolutions of the Walsh 2D masks. (**a**) The reconstructed images at 20; 40; 60; 80 and $100\%$ image reconstruction time respectively, for $48 \times 48$ pixel Walsh 2D masks. Followed by the corresponding enhanced images from the auto-encoder output. The confidence level, predicted by the recognition algorithm after enhancement, only for digit 9 is shown beneath. (**b**) Similarly for $96 \times 96$ pixel Walsh 2D masks. The solid black lines show the imposed stopping point for each case at around $20\%$ of image acquisition time.

Apart from testing object discrimination and mask types, we tested our technique robustness on different resolutions. Figure 6 shows the results obtained from the two-step approach for an image reconstructed by $48\times 48$ pixel (Fig. 6a) and $96\times 96$ pixel (Fig. 6b) resolution Walsh 2D masks. The images shown are the raw reconstructed images (top row), the auto-encoder enhanced images (middle row) and the corresponding confidence level for this particular object (bottom row). Testing different mask resolutions significantly changed the reconstruction time needed for each resolution. For the Walsh 2D masks a complete set scales as $N^2$, in Fig. 6 we show that although there is a significant increase in the number of masks required to image a 96 $\times$ 96 pixel object our two-step approach can be used to establish an early stopping point at 19% of the total image reconstruction time. That is, as the image reconstruction time increases we are still able to stop the experiment, with a $75\%$ confidence in image recognition, at $19\%$ of the reconstruction time. Furthermore, when applying this approach to real-time ghost imaging, by calculating the confidence average over an interval of five iterations it becomes possible to monitor in real-time the confidence increase and stop the experiment early.

## Conclusion

To summarise, we designed and implemented a two-step deep learning approach to establish an optimal early stopping point for ghost imaging experiments. We tested this approach on a non-degenerate ghost imaging setup while varying physical parameters, namely the mask type and resolution. After building, training and optimising our neural networks individually, we went through the reconstructed images after each mask to determine the optimal early stopping point. Training the networks as a pre-measurement step allows for instantaneous image enhancement and recognition post-measurement implying that this two-step approach can be implemented as a real-time early stopping technique. After imposing a strict evaluation criteria we determined that a recognition algorithm, individually, allows for the reduction of image reconstruction time to around 40% of the total reconstruction time. Adding in a two-step approach allows an even further drop in acquisition time to approximately 20% of the total reconstruction time. We tested the technique’s robustness to different mask types and different mask resolutions. While the technique is robust enough to handle a variation in both parameters it is also important to note that the Walsh 2D masks converge faster than the random masks and therefore the optimal point is reached quicker. We have shown that by employing this two-step approach we can establish an optimal early stopping point thereby reducing the number of measurements required by up to 80%, or a fivefold decrease in time and number of photons needed for image recognition. We see this technique as an efficient method of early stopping which can be implemented to establish real-time early stopping for various mask types and resolutions. While we demonstrated our method for amplitude-only objects, we anticipate it could be used other types of objects, such as phase-objects imaged through traditional quantum ghost imaging^38^, as well as in holographic imaging approaches used to extract the phase information of an object^39^, which will also benefit from reducing image reconstruction time. We, therefore, conclude that by using this approach the number of measurements required is significantly reduced while image information is preserved through enhancement and recognition. This, therefore, leads to a faster, more efficient image acquisition technique. We believe that this two-step deep learning approach will prove valuable to the community who are focusing their efforts on real-time ghost imaging.
